# From Lockdowns to Long COVID—Unraveling the Link Between Sleep, Chronotype, and Long COVID Symptoms

**DOI:** 10.3390/brainsci15080800

**Published:** 2025-07-28

**Authors:** Mariam Tsaava, Tamar Basishvili, Irine Sakhelashvili, Marine Eliozishvili, Nikoloz Oniani, Nani Lortkipanidze, Maria Tarielashvili, Lali Khoshtaria, Nato Darchia

**Affiliations:** 1 Tengiz Oniani Laboratory of Sleep-Wakefulness Cycle Study, Ilia State University, Tbilisi 0179, Georgia; mariam.tsaava.1@iliauni.edu.ge (M.T.); tamari.basishvili@iliauni.edu.ge (T.B.); marine.eliozishvili@iliauni.edu.ge (M.E.); nikoloz.oniani@iliauni.edu.ge (N.O.); nani.lortkipanidze@iliauni.edu.ge (N.L.); maria.tarielashvili.1@iliauni.edu.ge (M.T.); lali.khoshtaria.1@iliauni.edu.ge (L.K.); 2 Medical School, Georgian American University, Tbilisi 0160, Georgia; irinesakhelashvili@gau.edu.ge

**Keywords:** long COVID, COVID-19, sleep quality, chronotype, pre-sleep arousal

## Abstract

**Background/Objectives**: Given the heterogeneous nature of long COVID, its treatment and management remain challenging. This study aimed to investigate whether poor pre-pandemic sleep quality, its deterioration during the peak of the pandemic, and circadian preference increase the risk of long COVID symptoms. **Methods**: An online survey was conducted between 9 October and 12 December 2022, with 384 participants who had recovered from COVID-19 at least three months prior to data collection. Participants were categorized based on the presence of at least one long COVID symptom. Logistic regression models assessed associations between sleep-related variables and long COVID symptoms. **Results**: Participants with long COVID symptoms reported significantly poorer sleep quality, higher perceived stress, greater somatic and cognitive pre-sleep arousal, and elevated levels of post-traumatic stress symptoms, anxiety, depression, and aggression. Fatigue (39.8%) and memory problems (37.0%) were the most common long COVID symptoms. Sleep deterioration during the pandemic peak was reported by 34.6% of respondents. Pre-pandemic poor sleep quality, its deterioration during the pandemic, and poor sleep at the time of the survey were all significantly associated with long COVID. An extreme morning chronotype consistently predicted long COVID symptoms across all models, while an extreme evening chronotype was predictive only when accounting for sleep quality changes during the pandemic. COVID-19 frequency, severity, financial impact, and somatic pre-sleep arousal were significant predictors in all models. **Conclusions**: Poor sleep quality before the pandemic and its worsening during the pandemic peak are associated with a higher likelihood of long COVID symptoms. These findings underscore the need to monitor sleep health during pandemics and similar global events to help identify at-risk individuals and mitigate long-term health consequences, with important clinical and societal implications.

## 1. Introduction

The coronavirus disease 2019 (COVID-19) pandemic, along with social restrictions, financial instability, uncertainty, confinement, and other actions put in place, disrupted nearly every aspect of life around the globe. These unprecedented transformations in daily activities, feelings of insecurity and helplessness, in addition to virus-related health challenges, had major direct and indirect impacts on sleep and neuropsychiatric well-being. Since the progression of the pandemic, a large number of studies have reported high rates of various sleep and mental health problems worldwide at various stages of the pandemic. The prevalence of those problems has been reported to differ across different age ranges [1,2,3,4].

As the end of the pandemic was approaching, there was growing evidence that the burden of neuropsychiatric and/or physical impairments of COVID-19 may persist for a long time, beyond the acute disease period. The phenomenon of post-acute sequelae of COVID-19, known as long COVID, develops 3 months after symptom onset and persists for at least 2 months thereafter, and cannot be explained by alternative diagnoses [5]. Long COVID symptoms may persist from the initial stage of the disease, emerge after recovery, and fluctuate or relapse over time [5]. Long COVID covers a broad range of symptoms, among which fatigue, headache, cognitive impairments, shortness of breath, sleep disturbances, anxiety, depression, and post-traumatic stress disorder (PTSD) are considered to be its common manifestations [6,7].

Sleep disturbances are at the core of the post-acute sequelae of COVID-19 [8]. The prevalence of sleep disturbances in post-COVID condition varies widely, but a high rate of 77.7% has also been reported [9]. Given the key role that sleep plays in psychological and physical well-being, including immunological health, sleep dysfunction bears importance not only as a long COVID symptom but also as a factor potentially contributing to the incidence of long COVID. To date, only few studies have addressed the associations of pre-pandemic and pre-infection sleep problems with long COVID manifestation. Findings support the predictive role of pre-existing sleep problems in the development of post-COVID condition [10]. There is evidence of bidirectional association between pre-pandemic insomnia and long COVID [11]. It has also been reported that pre-infection sleep quality, sleep duration, and insomnia severity show a dose-dependent association with the number of symptoms at one/three months from COVID-19 infection [12,13]. Goncalves and colleagues found that individuals with long COVID symptoms had poorer self-perceived sleep quality in the month prior to their COVID-19 diagnosis [13]. However, due to the broad spectrum and heterogeneity of long COVID symptoms, our understanding of how pre-pandemic sleep quality and pandemic-related changes in self-reported sleep parameters may predispose individuals to long COVID remains limited.

Studies investigating the association between chronotype and COVID-19 infection consistently report that evening chronotypes show more evident changes in sleep and mental health, as well as a higher likelihood of being infected [14,15,16]. However, a higher rate of infection and hospitalization in morning chronotypes has also been reported [17]. As regards chronotype-based differences in long COVID, data are scarce. One study examining the association of chronotype with a greater risk of long COVID found that morning chronotype, assessed as one component of pre-infection multidimensional sleep health, was protective against post-COVID condition [18].

The present study aimed to investigate whether COVID-19 patients with poor sleep quality in the pre-pandemic period and worsening of sleep quality at the severe stage of the COVID-19 pandemic were at greater risk for the occurrence of long COVID symptoms. The association of circadian preference/chronotype with long COVID was also assessed. We hypothesize that poor pre-pandemic sleep quality and its deterioration during the peak of the pandemic are both associated with long COVID symptoms. We also hypothesize that individuals with extreme chronotypes are more likely to experience long COVID symptoms. The extent to which pre-pandemic sleep quality, pandemic-related changes, sleep quality at the time of the survey, and circadian preference predict long COVID symptoms will be examined.

## 2. Materials and Methods

### 2.1. Study Design and Participants

A cross-sectional online survey was conducted among the general population of Georgia, aged 18 years or older, between 9 October and 12 December 2022. Georgia experienced multiple waves of the COVID-19 pandemic, implementing lockdown measures of varying severity. COVID-19 vaccination in Georgia began on 15 March 2021. By 2022, Georgia had significantly relaxed restrictions, including lifting outdoor mask mandates and quarantine requirements. Despite occasional case surges, major restrictions have not been reinstated since March 2022 [19]. The most severe restrictions, along with a high number of cases and lack of vaccine availability, occurred during the critical stage of the pandemic from late 2020 to early 2021, hereafter referred to as the peak of the pandemic.

The survey was administered using a Google form hosted by Ilia State University, Georgia. The study was conducted in accordance with the Declaration of Helsinki and the study protocol was approved by the Ilia State University Research Ethics Committee (#140-35/1). Participation was voluntary and anonymous, and completing the questionnaire was considered as providing informed consent. From a total of 507 participants surveyed, 384 valid profiles of those who had recovered from COVID-19 more than 3 months prior to data collection were analyzed. The remaining participants were excluded from the analysis because they either had not been infected with COVID-19, had recovered less than three months before the assessment, or had invalid profiles (e.g., total sleep time exceeding time in bed). The age range of study participants was 18–70 years.

### 2.2. Measures

The structured questionnaire was similar to that previously administered [20,21] and consisted of 6 subsections. The first subsection collected socio-demographic data such as age, sex, marital status, education, employment status, and information on health status including chronic disease, body mass index (BMI), COVID-19 disease frequency (categorized as once or more than once) and severity (categorized as mild or severe), vaccination status, and COVID-19 impact on financial situation (no impact, slight impact, and strong impact). Of the infected participants, 82.8% were polymerase chain reaction (PCR)-confirmed, while 17.2% were probable cases based on symptoms and close contact with a confirmed case, most often a family member. All participants were categorized into a single COVID-19-infected group, as no differences in the number of long COVID symptoms or sleep quality measures were identified.

The following validated questionnaires, comprising the next four subsections, have been described in detail in earlier publications [20,21] and are briefly summarized below:

- Insomnia Severity Index (ISI) [22]—results from this measure are not reported in the current publication.
- Pittsburgh Sleep Quality Index (PSQI)—a PSQI score higher than 5 is widely recognized as indicative of poor sleep quality [23]. Cronbach’s α for the PSQI was 0.84 in the current study.
- Perceived Stress Scale-4 (PSS-4)—higher scores indicate higher levels of perceived stress [24,25]. Cronbach’s α for the scale was 0.76.
- Pre-Sleep Arousal Scale (PSAS) [26]—assesses symptoms of cognitive and somatic arousal experienced at bedtime, with clinically relevant cut-off scores of ≥14 for somatic arousal and ≥20 for cognitive arousal [27,28]. Cronbach’s α for the somatic subscale was 0.82, and for the cognitive subscale, −0.91.

Georgian versions of the study instruments (PSQI, PSS, PSAS) demonstrated good psychometric properties in the Georgian population, with details provided elsewhere [29,30,31].

The last subsection included a retrospective assessment of sleep quality (good, intermediate, or poor) before the pandemic. In addition, respondents were asked to rate changes in self-perceived sleep quality (no change, improvement, or worsening) during the peak of the pandemic (late 2020–early 2021).

Post-traumatic stress symptoms (PTSS) were assessed using the Abbreviated PTSD Checklist (PCL2), which evaluates the experience of repeated disturbing thoughts about stressful events in the past and feeling very upset when recalling them [32]. Both questions are rated on a five-point scale (1 to 5), and a sum score ≥4 is indicative of PTSS.

Chronotype was assessed by asking respondents to identify their circadian preference from the following response alternatives: (a) very alert/active in the morning and sleepy early in the evening (extreme morning-type), (b) moderately alert in the morning and sleepy in the evening (moderately morning-type), (c) neither morning nor evening person (intermediate-type), (d) moderately alert in the evening and sleepy in the morning (moderately evening-type), (e) very alert/active in the evening and sleepy in the morning (extreme evening-type) [33].

Finally, respondents were presented with a list of 9 long COVID symptoms: fatigue, deterioration of smell or taste, memory problems, fever, headache, joint/muscle pain, chest pain, coughing, and shortness of breath. They were instructed to indicate the presence of these symptoms only if they met the WHO criteria for long-term symptoms—that is, persisting or newly developed symptoms occurring 3 months after the initial COVID-19 infection, lasting for at least 2 months and not explained by alternative diagnoses [5].

### 2.3. Statistical Analysis

Demographic, sleep, health, and psychosocial variables are presented as means ± standard deviations (SDs) for continuous variables and as counts and percentages for categorical variables. Variables were assessed for assumptions required for parametric and nonparametric tests and analyzed accordingly. Between-group comparisons were conducted using chi-square tests for categorical variables and independent-samples *t*-tests for continuous variables.

A broad spectrum of psychiatric and psychobehavioral indices, including depression, anxiety, and PTSD, have been identified as long COVID symptoms and/or as predisposing factors for experiencing long-term symptoms after COVID-19 [34]. In this survey, mental health symptoms were not included in the list of long COVID symptoms, making it difficult to differentiate them from possible pre-existing conditions. Instead, for regression analyses, scores for anxiety, depression, PTSS, aggressive behavior, and feelings of social isolation, each measured on the same scale, were summed to create a composite variable representing psychobehavioral symptoms, and the association of this composite variable with the occurrence of long COVID symptoms was tested.

To explore predictors of long COVID symptoms, respondents were categorized into two groups: those without any symptoms and those reporting at least one long COVID symptom. Binary logistic regression analysis was conducted with group status (long COVID vs. non-long COVID) as the dependent variable. COVID-19-related and sleep variables were included as independent variables. Models were adjusted for age, sex, marital status, education, employment status, chronic disease, BMI, psychobehavioral symptoms, and perceived stress. Model 1 included COVID-19-related and sleep variables measured at the time of the survey as predictors of long COVID symptoms. Model 2 added subjective sleep quality before the pandemic as an additional predictor. Model 3 tested changes in sleep quality during the severe pandemic period, in addition to predictors in Model 1.

All categorical variables were dummy coded, and all regression models were checked for multicollinearity using the variance inflation factor (VIF), which was below 2.14 for all variables. A two-tailed significance level was set at 0.05. The statistical analyses were performed using the Statistical Package for Social Sciences (SPSS) version 22.0.

## 3. Results

### 3.1. Sample Characteristics

**Figure 1:**
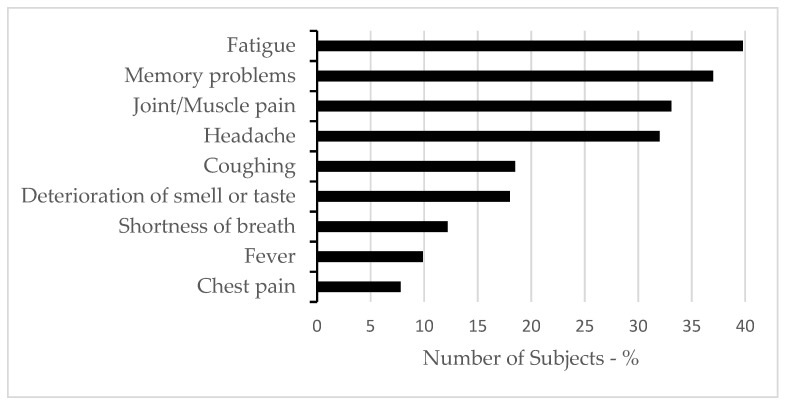
Self-reported prevalence of long COVID symptoms.

The study included a total of 384 participants infected with the SARS-CoV-2 virus. Among them, 74% reported having at least one long COVID symptom. Consequently, based on the criteria described in Section 2.2, these participants formed the long COVID group. The prevalence and types of long COVID symptoms are presented in Figure 1. Out of the nine symptoms assessed, fatigue and memory problems were the most commonly reported, by 39.8% and 37.0% of subjects, respectively.

Table 1 presents the characteristics of subjects according to the presence or absence of long COVID symptoms. Compared with subjects without long COVID symptoms, those with at least one long COVID symptom were more likely to report poor sleep quality (*p* < 0.001), higher levels of perceived stress (*p* = 0.036) and post-traumatic stress symptoms (*p* = 0.010), clinically relevant somatic and cognitive pre-sleep arousal (*p* < 0.001 for both), as well as higher levels of anxiety (*p* = 0.002), depression (*p* < 0.001), and aggressive behavior (*p* = 0.002). There were no statistically significant differences between groups in age, gender, marital status, education, BMI, chronic disease, and level of social isolation. However, the frequency of infection (*p* = 0.001), illness severity (*p* = 0.001), and the impact of COVID-19 on financial situation (*p* = 0.002) were greater, while the vaccination rate (*p* = 0.027) was lower among subjects with long COVID symptoms.

### 3.2. Sleep Variables

The mean PSQI global score was 6.20 ± 3.8 (mean ± SD). At the time of the survey, the prevalence of poor sleepers was 45.8%. The difference in PSQI scores between subjects with and without long COVID symptoms was statistically significant (6.85 vs. 4.34, *p* < 0.001). More than half (53.5%) of participants with long COVID symptoms were poor sleepers (PSQI > 5), compared to 24.0% of those without symptoms. Further, the long COVID group exhibited significantly worse outcomes across all PSQI measures, including sleep disturbances (*p* = 0.001), subjective sleep quality (*p* < 0.001), daytime dysfunction (*p* < 0.001), sleep latency (*p* < 0.001), sleep efficiency (*p* = 0.004), use of sleeping medication (*p* < 0.001), and sleep duration (*p* < 0.001). In addition, the PSQI global score differed significantly between respondents with mild and severe COVID-19 infection (5.75 vs. 6.56, *p* = 0.040).

According to subjective evaluation, 58.6% of participants classified their sleep quality before the pandemic as good, 29.4% as intermediate, and 12.0% as poor. During the peak of the pandemic, 34.6% of participants reported a worsening of sleep quality compared to the pre-pandemic period, while 60.7% reported no change, and 4.7% experienced an improvement. Significant differences were found between participants with and without long COVID symptoms in both pre-pandemic sleep quality (*p* = 0.015) and changes in sleep quality during the pandemic peak (*p* < 0.001).

### 3.3. Associations Between Sleep and Long COVID Symptoms

To address the role of sleep variables in predicting long COVID symptoms, multiple logistic regression analyses were conducted. All models were adjusted for socio-demographic and health variables such as age, gender, marital status, education level, employment status, chronic disease, BMI, perceived stress, and psychobehavioral symptoms. Model 1 tested pandemic-associated variables and sleep variables assessed at the time of the survey. Significant predictors of long COVID symptoms in this model included pandemic-related variables, among which the impact of the pandemic on financial situation showed the highest predictive power (Exp(B) = 2.433, *p* = 0.010). Additionally, both the frequency and severity of COVID-19 infection were significant predictors. In contrast, vaccination status was not associated with long COVID symptoms. With regard to sleep variables, sleep quality assessed by the PSQI at the time of the survey, extreme morning chronotype, and somatic pre-sleep arousal significantly predicted long-term symptoms (Table 2). Of all variables, poor sleep quality demonstrated the highest predictive power (Exp(B) = 2.709, *p* = 0.001).

Model 2 tested subjective pre-pandemic sleep quality in addition to the variables included in Model 1. We observed that poor sleep quality prior to the pandemic was a significant predictor of long COVID symptoms. Furthermore, additional analyses that replaced pre-pandemic sleep quality with a variable assessing changes in sleep quality during the peak of the pandemic (Model 3) revealed that worsening of sleep quality during this critical period had the strongest predictive power for long COVID symptoms (Exp(B) = 3.164, *p* = 0.001).

Somatic pre-sleep arousal and an extreme morning chronotype remained significant predictors across all models. The extreme evening chronotype became significant (*p* < 0.05) in Model 3. Regarding COVID-19-related variables, infection severity, frequency of illness, and the financial impact of the pandemic were consistently associated with long COVID symptoms in all models. All models were statistically significant (*p* < 0.01) and correctly classified roughly 80% of cases.

## 4. Discussion

A large number of studies have reported long COVID symptoms affecting health and normal quality of life worldwide [6,35]. The evolution of long COVID has rapidly become a major public health concern. We also observed a high prevalence of individuals (74%) with at least one self-reported long COVID symptom. Our finding that fatigue and memory problems were the most commonly reported long-lasting symptoms aligns with other reports [36,37]. A comprehensive description of sleep and mental health among individuals with and without long COVID symptoms revealed differences consistent with previous studies [1,8]. Symptoms of anxiety, depression, aggressive behavior, feeling socially isolated, PTSS, and perceived stress all differed significantly between groups. Similarly, we observed a high prevalence of poor sleep quality among individuals with long COVID symptoms. An overview of PSQI sub-components showed that the long COVID group exhibited significantly worse outcomes across all dimensions of sleep quality measured by PSQI.

The prevalence of poor sleep quality at the time of the survey in the total sample was 45.8%. Although this prevalence rate is lower compared to an earlier survey conducted during the initial months of the COVID-19 outbreak in Georgia [20], our findings align with evidence from most studies suggesting a prospective long-term effect of COVID-19 on sleep [37,38,39].

We hypothesized that poor sleep quality before the pandemic and worsening sleep quality during the peak of the pandemic would be significantly associated with long COVID symptoms. We found that not only sleep quality measured by PSQI at the time of the survey predicted long COVID symptoms, but retrospectively assessed pre-pandemic poor sleep quality was also a significant predictor, even after adjusting for demographic and health variables. Interestingly, when we assessed changes in sleep quality during the peak of the pandemic, we found that the predictive power of worsening sleep quality was the highest among all variables in the model. A recent study [10] reported the significant association of self-reported pre-existing sleep problems with the risk of developing post-COVID-19 syndrome in a large number of participants. The association of post-COVID condition with pre-pandemic or pre-infection sleep quality, as well as changes in sleep health (duration, quality, disturbances), has also been observed [11,12,13].

Evidence from a limited number of studies addressing the role of sleep in long COVID indicates that sleep contributes to a greater risk of developing long COVID [10,11,12,13]. Although we cannot differentiate whether current sleep problems are newly appeared or pre-existing, we found that the risk of developing long COVID symptoms was associated with poor sleep quality at all three time points. Our findings show that current sleep quality, as well as retrospective reports of both pre-pandemic sleep quality and worsening of sleep quality during the peak of the pandemic, increase the risk of long COVID symptoms. These findings, along with existing research on this issue, underscore the importance of improved clinical management of sleep problems in the context of long COVID. Prevention and treatment of long COVID syndrome require a more systematic approach to sleep assessment. The impact of sleep on the progression of long COVID, as well as other post-viral syndromes, should be recognized. Moreover, the healthcare sector should acknowledge that sleep quality, and its worsening under the impact of major events such as pandemics, may predispose individuals to subsequent long-term health complications.

A second noteworthy finding of the study is that chronotype also contributes to long COVID symptoms. In our study, an extreme morning chronotype was a significant predictor of long COVID symptoms in all models. Accounting for changes in sleep quality during the pandemic peak revealed an association with an extreme evening chronotype as well. Particular vulnerability of evening-type individuals to sleep disturbances and psychological problems was found in a number of studies [40,41]. While increased flexibility of social schedules and reduced social jetlag have been reported to increase sleep duration [42,43,44], changes in sleep and lifestyle behavior during the pandemic have been shown to decrease sleep quality [21,45]. Considering that multiple waves of pandemic lockdowns and the loss of habitual daily routines, along with follow-up waves of resumptions and further loss of acquired daily habits, the progressive disruption of sleep and circadian rhythms since the start of the pandemic is not surprising [15,44,45]. Ultimately, such disruptions may lead to dysfunction of the immune system and overall health [46,47]. Adjustments to the delay in sleep phase under the lockdowns [35,36] as well as to changes imposed during re-openings were most likely more challenging for extreme chronotypes, as hypothesized, thereby increasing their vulnerability for developing long COVID symptoms.

As expected, disease severity, frequency, and impact on financial situation were significantly associated with long COVID symptoms in all models, whereas vaccination status was not. Although the number of symptoms was lower among vaccinated respondents, the difference was not significant (2.4 vs. 2.0, *p* = 0.081). The most likely explanation for this result is the high vaccination rate (74.7%) among respondents and the relatively stable epidemic situation in the country at the time of the survey [19]. The impact of financial situation likely affected the availability of healthcare support, which, in turn, may have had consequences in the long run. Somatic pre-sleep arousal was one of the variables consistently associated with long COVID. We have previously reported the role of pre-sleep arousal for sleep and mental health [20,21,31]. The association of somatic pre-sleep arousal with long COVID symptoms has not been previously reported. This finding suggests that assessing distinct features of sleep behavior during pandemics, and more broadly during post-viral conditions, could be beneficial in preventing long-term health complications.

Given the crucial role of sleep and circadian rhythms in regulating the immune system, poor sleep quality and short sleep duration have been linked to increased vulnerability to viral infections [46,48]. Stress and personal vulnerability to stressful events also play a major role in predisposing individuals to sleep disturbances [29,49,50]. Based on this evidence, the increased need for continued COVID-19 research is still warranted, as promoting sleep health during disease outbreaks could offer significant public health and societal benefits.

This study has some limitations. First, the cross-sectional design prevents the establishment of causal relationships between sleep disturbances and long COVID symptoms. Second, there might be a recruitment bias due to the online survey, potentially excluding individuals with limited internet access. Third, reliance on self-reported data may introduce some recall bias, particularly regarding pre-pandemic sleep quality. Furthermore, we assessed a limited number of long COVID symptoms, which may have limited our ability to conduct a more comprehensive analysis of the observed associations. Finally, the high proportion of female participants (85.2%) in this study, consistent with many online COVID-19 surveys, could have affected the observed rates of poor sleep quality and long COVID symptoms.

## 5. Conclusions

Due to the complex and heterogeneous nature of long COVID, its etiology remains poorly understood, making treatment and management particularly challenging. This study supports previous findings linking pre-pandemic sleep to long COVID risk and expands the existing body of evidence by highlighting the impact of changes in sleep quality during the severe pandemic period. The results suggest that: (1) respondents with poor pre-pandemic sleep quality had a higher risk of developing long COVID symptoms; (2) worsening of sleep quality during pandemics, and likely similar global events, along with the impact of uncertainty, stress, and lifestyle changes associated with such events, can have long-lasting effects on health; and (3) extreme chronotypes are more sensitive to alterations in sleep behavior, with an increased risk for health problems. Monitoring sleep health during such periods may help identify high-risk groups for targeted interventions, with important clinical and societal implications.

## Figures and Tables

**Table 1:** Demographic, health, and psychosocial variables of the whole sample and separately by the presence of long COVID symptoms.

	Total Sample n = 384	Long COVID n = 284	Non-Long COVID n = 100	Statistics
**Age**	41.76 ± 12.8	41.42 ± 12.66	42.71 ± 13.38	t_(382)_ = 0.864, *p* = 0.388
**Sex**				
Male	57 (14.8%)	42 (14.8%)	15 (15.0%)	χ^2^(1) = 0.003, *p* = 0.959
Female	327 (85.2%)	242 (85.2%)	85 (85.0%)	
**Marital status**				
Married/cohabiting	225 (58.6%)	167 (58.8%)	58 (58.0%)	χ^2^(1) = 0.020, *p* = 0.889
Single/divorced/widowed	159 (41.4%)	117 (41.2%)	42 (42.0%)	
**Education**				
University	323 (84.1%)	237 (83.5%)	86 (86.0%)	χ^2^(1) = 0.360, *p* = 0.549
High school/student	61 (15.9%)	47 (16.5%)	14 (14.0%)	
**Employment**				
Yes	299 (77.9%)	220 (77.5%)	79 (79.0%)	χ^2^(1) = 0.101, *p* = 0.750
No	85 (22.1%)	64 (22.5%)	21 (21.0%)	
**Chronic disease**				
Yes	70 (18.2%)	51 (18.0%)	19 (19.0%)	χ^2^(1) = 0.054, *p* = 0.816
No	314 (81.8%)	233 (82.0%)	81 (81.0%)	
**COVID-19 Frequency**				
Once	242 (63.0%)	165 (58.1%)	77 (77.0%)	χ^2^(1) = 11.338, *p* = **0.001**
≥1	142 (37.0%)	119 (41.9%)	23 (23.0%)	
**COVID-19 Severity**				
Mild	170 (44.3%)	112 (39.4%)	58 (58.0%)	χ^2^(1) = 10.330, *p* = **0.001**
Severe	214 (55.7%)	172 (60.6%)	42 (42.0%)	
**COVID-19 Impact on Finances**				
No impact	114 (29.7%)	73 (25.7%)	41 (41.0%)	χ^2^(2) = 12.127, *p* = **0.002**
Slight impact	137 (35.7%)	100 (35.2%)	37 (37.0%)	
Strong impact	133 (34.6%)	111 (39.1%)	22 (22.0%)	
**Vaccination**				
Yes	287 (74.7%)	204 (71.8%)	83 (83.0%)	χ^2^(1) = 4.887, *p* = **0.027**
No	97 (25.3%)	80 (28.2%)	17 (17.0%)	
**BMI**	25.6 ± 4.9	25.60 ± 4.90	25.67 ± 4.91	t_(382)_ = 0.112, *p *= 0.911
**PSS**	6.6 ± 2.5	6.77 ± 2.53	6.15 ± 2.50	t_(382)_ = −2.107, *p* **< 0.036**
**Anxiety**	2.90 ± 1.23	3.01 ± 1.24	2.57 ± 1.17	t_(382)_ = −3.136, *p* **= 0.002**
**Depression**	2.69 ± 1.34	2.86 ± 1.32	2.21 ± 1.26	t_(382)_ = −4.277, *p* **< 0.001**
**Aggression**	2.32 ± 1.14	2.42 ± 1.16	2.02 ± 1.04	t_(382)_ = −3.065, *p* **= 0.002**
**PTSS**	4.03 ± 2.14	1.94 ± 1.12	1.61 ± 0.97	t_(382)_ = −2.598, *p* **= 0.010**
**Social Isolation**	2.10 ± 1.16	2.18 ± 1.17	1.87 ± 1.08	t_(382)_ = −2.290, *p* **= 0.023**
**Chronotype**				
Extreme morning	90 (23.4%)	69 (24.3%)	21 (21.0%)	χ^2^(4) = 6.485, *p* = 0.166
Morning	80 (20.8%)	57 (20.1%)	23 (23.0%)	
Intermediate	75 (19.5%)	48 (16.9%)	27 (27.0%)	
Evening	79 (20.6%)	62 (21.8%)	17 (17.0%)	
Extreme evening	60 (15.6%)	48 (16.9%)	12 (12.0%)	
**Clinically Relevant PSAS- ** **Somatic**				
Yes	162 (42.2%)	139 (48.9%)	23 (23.0%)	χ^2^(1) = 20.410, *p* < **0.001**
No	222 (57.8%)	145 (51.1%)	77 (77.0%)	
**Clinically Relevant PSAS- ** **Cognitive**				
Yes	186 (48.4%)	154 (54.2%)	32 (32.0%)	χ^2^(1) = 14.627, *p* < **0.001**
No	198 (51.6%)	130 (45.8%)	68 (68.0%)	
**PSQI score**	6.2 ± 3.8	6.85 ± 0.2	4.34 ± 0.3	t_(382)_ = −5.876, *p* < **0.001**
**PSQI categories**				
Poor	176 (45.8%)	152 (53.5%)	24 (24.0%)	χ^2^(1) = 25.962, *p* < **0.001**
Good	208 (54.2%)	132 (46.5%)	76 (76.0%)	
**Pre-Pandemic Sleep Quality**				
Good	225 (58.6%)	159 (56.0%)	66 (66.0%)	χ^2^(2) = 8.467, *p* = **0.015**
Intermediate	113 (29.4%)	83 (29.2%)	30 (30.0%)	
Poor	46 (12.0%)	42 (14.8%)	4 (4.0%)	
**Changes in Sleep Quality During the Pandemic Peak**				
No change	233 (60.7%)	154 (54.2%)	79 (79.0%)	χ^2^(2) = 19.759, *p* < **0.001**
Worsening	133 (34.6%)	116 (40.9%)	17 (17.0%)	
Improving	18 (4.7%)	14 (4.9%)	4 (4.0%)	

Data are presented as the mean and standard deviation or counts and percentages. Note. BMI, body mass index; PSS, Perceived Stress Scale; PTSS, post-traumatic stress symptoms; PSAS-Somatic, Somatic Pre-Sleep Arousal; PSAS-Cognitive, Cognitive Pre-Sleep Arousal; PSQI, Pittsburgh Sleep Quality Index. Variable names and statistically significant differences appear in bold.

**Table 2:** Prediction of long COVID symptoms based on the logistic regression models.

Predictor		Model 1		Model 2		Model 3	
		OR (95% CI)	*p*	OR (95% CI)	*p*	OR (95% CI)	*p*
**COVID-19 Frequency**							
Once		Reference		Reference		Reference	
≥1		2.42 (1.36–4.31)	**0.003**	2.68 (1.49–4.82)	**0.001**	2.04 (1.13–3.67)	**0.018**
**COVID-19 Severity**							
Mild		Reference		Reference		Reference	
Severe		2.03 (1.20–3.44)	**0.008**	2.18 (1.27–3.75)	**0.005**	2.00 (1.16–3.43)	**0.012**
**COVID-19 Impact on Finances**							
No impact		Reference		Reference		Reference	
Slight impact		1.39 (0.74–2.61)	0.300	1.51 (0.80–2.86)	0.204	1.35 (0.71–2.57)	0.358
Strong impact		2.43 (1.24–4.78)	**0.010**	2.49 (1.25–4.95)	**0.009**	2.46 (1.23–4.91)	**0.011**
**Vaccination**							
No		Reference		Reference		Reference	
Yes		1.70 (0.84–3.46)	0.143	1.61 (0.78–3.34)	0.201	1.75 (0.84–3.63)	0.135
**Sleep Quality**							
Good		Reference		Reference		Reference	
Poor		2.71 (1.47–4.99)	**0.001**	3.02 (1.57–5.81)	**0.001**	2.48 (1.34–4.60)	**0.004**
**Chronotype**							
Intermediate		Reference		Reference		Reference	
Extreme morning		2.63 (1.20–5.76)	**0.016**	2.59 (1.17–5.75)	**0.019**	2.71 (1.22–6.04)	**0.015**
Morning		1.54 (0.70–3.35)	0.282	1.57 (0.71–3.48)	0.266	1.62 (0.72–3.62)	0.244
Evening		1.89 (0.84–4.26)	0.124	1.99 (0.86–4.57)	0.107	1.90 (0.83–4.35)	0.128
Extreme evening		2.23 (0.89–5.60)	0.087	2.12 (0.83–5.39)	0.115	2.82 (1.09–7.29)	0.033
**Clinically Relevant PSAS- ** **Somatic**							
No		Reference		Reference		Reference	
Yes		1.97 (1.01–3.87)	**0.049**	2.44 (1.20–4.96)	**0.013**	2.13 (1.06–4.25)	**0.033**
**Clinically Relevant PSAS- ** **Cognitive**							
No		Reference		Reference		Reference	
Yes		1.24 (0.63–2.44)	0.535	1.08 (0.54–2.16)	0.834	1.34 (0.67–2.70)	0.405
**Pre-Pandemic Sleep Quality**							
Good				Reference			
Intermediate				1.86 (0.56–6.12)	0.308		
Poor				0.42 (0.21–0.82)	**0.011**		
**Changes in Sleep Quality During the Pandemic Peak**							
No change						Reference	
Worsening						3.16 (1.64–6.09)	**0.001**
Improving						0.92 (0.25–3.35)	0.901
**Nagelkerke R^2^**		0.258		0.290		0.300	
**Correct classification (%)**		77.6%		78.1%		78.9%	

All models were adjusted for age, sex, marital status, education level, employment status, chronic disease, BMI, perceived stress, and psychobehavioral symptoms. OR, odds ratio; CI, confidence interval; PSAS-Somatic, Somatic Pre-Sleep Arousal; PSAS-Cognitive, Cognitive Pre-Sleep Arousal. Variable names and statistically significant differences appear in bold.

## Data Availability

The data that support the findings of this study are available from the corresponding author upon reasonable request due to ethical/legal reasons.
